# Establishment of Protoplast Preparation and Genetic Transformation Methods in Two *Ilyonectria* Species

**DOI:** 10.3390/jof12070488

**Published:** 2026-07-02

**Authors:** Yaoyao Wang, Weiwei Zhang, Xiaohan Wang, Ximei Zhang, Xiaohong Lu, Xiu Wang, Weiwei Gao

**Affiliations:** 1 Institute of Medicinal Plant Development, Chinese Academy of Medical Sciences & Peking Union Medical College, Beijing 100193, China; wangyaoyao@implad.ac.cn (Y.W.); xhwang@implad.ac.cn (X.W.); xwang@implad.ac.cn (X.W.); 2 College of Plant Science and Technology, Beijing University of Agriculture, Beijing 102206, China; wwzhang@bua.edu.cn; 3 School of Biology and Wine Engineering, Taishan University, Taian 271000, China; zhangximei2008@163.com; 4 State Key Laboratory for Biology of Plant Diseases and Insect Pests, Institute of Plant Protection, Chinese Academy of Agricultural Sciences, Beijing 100193, China; luxiaohong@caas.cn

**Keywords:** *Ilyonectria robusta*, *I. vredehoekensis*, protoplast, PEG/CaCl_2_ transformation

## Abstract

*Ilyonectria* is a common soil-inhabiting fungal genus that comprises numerous plant phytopathogenic species capable of infecting a wide array of crops, medicinal herbs, and horticultural plants. However, the lack of a reliable and efficient genetic transformation method has severely hindered the elucidation of the pathogenic mechanisms of *Ilyonectria* pathogens. In this study, we established an efficient protoplast-mediated genetic transformation method for two dominant *Panax* root rot pathogens, *I. robusta* and *I. vredehoekensis*. Key parameters governing high-quality protoplast preparation, including mycelium culture time, enzyme composition, osmotic stabilizer type, digestion speed, and digestion time, were systematically optimized. Subsequently, orthogonal experiments were conducted to optimize the PEG-CaCl_2_-mediated transformation conditions and to screen regeneration media for protoplasts. The optimal enzymatic system is composed of 20 mg/mL driselase and 10 mg/mL lysing enzyme, with 0.7 M NaCl as the osmotic stabilizer. Under these conditions, high-viability and high-quality protoplasts were obtained from *I. vredehoekensis* after 3 h of digestion at 150 rpm, and from *I. robusta* after 2 h of digestion at 100 rpm, yielding 5.52 × 10^7^ CFU/mL and 5.75 × 10^7^ CFU/mL protoplasts, respectively. Efficient transformation was achieved using a mannitol-prepared STC buffer mediated by 40% PEG4000. PCR and fluorescence microscopy verified positive transformants. Additionally, pathogenicity assays showed no significant differences in virulence between the transformed and wild-type strains, suggesting that the transformation procedure did not alter virulence. To the best of our knowledge, this is the first study to successfully establish genetic transformation methods for *I. robusta* and *I. vredehoekensis*, providing an essential technical platform for functional gene analysis, pathogenicity studies, and host–pathogen interaction research. In addition, the optimized transformation strategy may serve as a valuable reference for studies on other *Ilyonectria* species.

## 1. Introduction

The genus *Ilyonectria* is a widely distributed soil-inhabiting fungus belonging to the family Nectriaceae, order Hypocreales, phylum Ascomycota, that causes severe root and stem diseases in a wide range of economic and medicinal plants worldwide, infecting over 70 host plant species and causing substantial economic losses [1,2,3,4,5]. It was separated from *Neonectria*, or its anamorphic genus *Cylindrocarpon*, in 2011 based on molecular systematic evidence and was recognized as an independent genus [6]. To date, a total of 36 *Ilyonectria* species have been taxonomically validated globally, with the nucleotide sequence data of 31 species publicly deposited in MycoBank [7] (https://www.mycobank.org, accessed on 30 May 2026) and Index Fungorum [8] (https://www.indexfungorum.org/names/Names.asp, accessed on 30 May 2026), Among them, *I. robusta* and *I. vredehoekensis* are important pathogens for a variety of plants [2,4,9].

*I. robusta* exhibits a global cosmopolitan distribution across all continents except Antarctica and infects more than 20 plant genera, triggering multiple destructive diseases, including black foot disease, root rot, and stem canker [2,3,5,10,11,12,13,14,15,16,17,18,19,20,21]. However, *I. vredehoekensis*, which is predominantly isolated and reported in Ecuador, South Africa, and China, displays a narrower host range and specific regional distribution, exclusively causing root rot in American ginseng (*Panax quinquefolius*), apple (*Malus pumila*), *Pinus taeda*, and other host plants [1,9,22,23]. As destructive soil-borne pathogens, both species seriously threaten the cultivation of medicinal and woody plants. Specifically, root rot caused by them is the most devastating disease affecting Panax medicinal herbs in China, with field disease incidence ranging from 9.8% to 41.0%, resulting in substantial losses in the commodity quality and medicinal efficacy of *Panax* herbs [1,5,18]. Although the basic biological characteristics of the two pathogens have been preliminarily documented [24], the pathogenic molecular mechanisms of *I. robusta* and *I. vredehoekensis* remain poorly understood, largely due to the lack of mature genetic manipulation systems for this fungal genus.

Genetic transformation is an indispensable technical foundation for investigating the molecular mechanisms of fungal pathogenicity. The commonly used techniques for filamentous fungal species mainly consist of protoplast-mediated transformation (PMT) [25,26], *Agrobacterium tumefaciens*-mediated transformation (ATMT) [27,28], and electroporation (EP) [29]. Among these approaches, ATMT and PMT are the most widely adopted. Compared with ATMT, PMT features simpler procedures and no reliance on expensive instruments [30]. The success of PMT is highly dependent on high-quality protoplast preparation, and key technical parameters, including mycelial culture time, enzyme composition, osmotic stabilizer type, shaking speed, and digestion duration, vary significantly among different fungal species due to divergent cell structures and physiological characteristics [31]. Subsequently, the cell membrane permeability is enhanced with the help of polyethylene glycol (PEG), facilitating the uptake of DNA-Ca^2+^ complexes into protoplasts. Relying on a suitable resistant regeneration medium, the cell wall re-covers, and positive transformants are obtained. At present, mature PMT methods have been successfully established and widely optimized for classic phytopathogenic fungi such as *Fusarium* [32], *Trichoderma* [33], and *Colletotrichum* [34], providing effective support for functional gene verification and research into their pathogenic mechanisms. However, no efficient and mature genetic transformation method has been reported for *Ilyonectria* species, which severely restricts in-depth study on their virulence genes and pathogenic molecular mechanisms.

Given the forementioned research gaps, this study systematically aimed to establish and optimize a stable PEG-mediated protoplast transformation method for *I. robusta* and *I. vredehoekensis*. Three core technical modules, including protoplast preparation conditions, protoplast regeneration medium, and PEG-mediated genetic transformation parameters were optimized. This study represents the first report of a successful genetic transformation method for *Ilyonectria* fungi. The established efficient transformation protocol fills the key technical gap in genetic manipulation of *Ilyonectria*-induced plant diseases and provides a reliable technical platform for subsequent functional verification of virulence-related genes, elucidation of molecular pathogenic mechanisms, and development of targeted prevention and control strategies.

## 2. Materials and Methods

### 2.1. Fungal Strains, Plasmid, and Reagents

#### 2.1.1. Fungal Strains

The fungal strains *I. robusta* and *I. vredehoekensis* used in this study were isolated from diseased American ginseng roots collected in Weihai, Shandong Province, China (37.2° N, 122.1° E). The identification and preservation of the two strains have been reported in previous studies [1,5], and the isolates were long-term preserved in our laboratory in 15% glycerol at −80 °C in an ultra-low-temperature freezer.

#### 2.1.2. Plasmid

The pCT74 expression vector plasmid (5.7 kb, Wuhan Miaoling Biotechnology, Wuhan, China) was used for fungal transformation. This plasmid carries the *ToxA* promoter, hygromycin resistance gene (*hyg*), and *GFP* gene. The *hyg* cassette functions as a selectable marker, and only transformants with stable plasmid integration survive on hygromycin-selective medium, thereby excluding untransformed wild-type strains. The *GFP* reporter enables confocal microscopy to confirm the presence of positive transformants and to trace the subcellular localization of target proteins.

#### 2.1.3. Reagents

All fungal strains were cultured on PDA medium at 20 °C. CMC liquid medium (27.5 g CMC powder per liter of distilled water) was used to induce conidiation. YEPD liquid medium (3 g yeast extract, 10 g peptone, 20 g dextrose per liter of distilled water) was used for mycelial culture. For protoplast regeneration, TB3 regeneration medium without antibiotics (3 g yeast extract, 3 g acid-hydrolyzed casein, 220 g mannitol, 9 g agar per liter of distilled water) was used. Transformants were screened on TB3 regeneration medium supplemented with 25 μg/mL hygromycin B.

To optimize the protoplast preparation system, 0.7 M NaCl (58.44 g NaCl per liter of distilled water), 1.2 M KCl (89.4 g KCl per liter of distilled water), and 1.2 M MgSO_4_ (295.76 g MgSO_4_·7H_2_O per liter of distilled water) were compared as osmotic stabilizers. To maintain the osmotic pressure of protoplasts and ensure transformation efficiency, three types of STC Buffer were prepared. STC buffer 1 (200 g mannitol, 7.352 g CaCl_2_·2H_2_O, 50 mL 1 M Tris-Cl (pH 8.0), adjusted to 1 L with distilled water), STC buffer 2 (200 g sucrose, 7.352 g CaCl_2_·2H_2_O, 50 mL 1 M Tris-Cl (pH 8.0), adjusted to 1 L with distilled water), and STC buffer 3 (200 g sorbitol, 7.352 g CaCl_2_·2H_2_O, 50 mL 1 M Tris-Cl (pH 8.0), adjusted to 1 L with distilled water). PTC buffer was prepared by dissolving PEG4000 in STC buffer at the optimal proportion and was used for PEG-mediated protoplast transformation.

Driselase and lysing enzyme were used to treat fungal cell walls during protoplast preparation; both reagents were commercially acquired from Sigma-Aldrich, St. Louis, MO, USA. Hygromycin B was purchased from Roche (Mannheim, Germany) and supplied by Beijing Yishan Huitong Technology (Beijing, China). The PDA powder, yeast extract, and peptone were obtained from OXOID Ltd. (Basingstoke, UK). CMC was from WGDF (Beijing, China); acid-hydrolyzed casein was from Biotopped; and mannitol, agar, and Tris-Cl were from Solarbio (Beijing, China). Dextrose, NaCl, KCl, MgSO_4_, CaCl_2_·2H_2_O, sucrose, sorbitol, and PEG4000 were purchased from Sinopharm Chemical Reagent Co., Ltd. (Shanghai, China). All PCR primers used in this study were synthesized by Beijing Liuhe Huada Gene Technology (Beijing, China).

### 2.2. Hygromycin Sensitivity Assay

To determine the optimal screening concentration of hygromycin B, the antibiotic was added to PDA medium to prepare selective plates at final concentrations of 0, 5, 10, 25, 50, and 100 μg/mL. Fungal strains were inoculated at the center of each plate and cultured at 20 °C for 14 days. Each treatment was performed in triplicate. Fungal growth was regularly observed and recorded to assess sensitivity to hygromycin B. The minimum concentration that completely inhibited fungal growth was defined as the selective concentration for subsequent transformation screening.

### 2.3. Protoplast Preparation

#### 2.3.1. Mycelial Culture Time

Fungal strains cultured on PDA were inoculated into CMC liquid medium and incubated at 20 °C with continuous shaking at 200 rpm for 7 d to induce abundant conidiation. The cultures were filtered through a double-layer sterile filter cloth, and the resulting filtrate was centrifuged for 10 min at 10,000 rpm. After discarding the supernatant, sterile distilled water was added to adjust the spore suspension to 10^7^ CFU/mL. The prepared spore suspension was transferred into YEPD liquid medium and cultured at 20 °C with shaking at 200 rpm. Mycelia were collected by filtration at 48 h, 72 h, and 96 h post-incubation, and then washed thoroughly with an osmotic stabilizer to remove residual medium. The enzyme solution consisted of 20 mg/mL driselase and 10 mg/mL lysing enzyme, prepared in 0.7 M NaCl. The mixed enzyme solution was pre-incubated at 30 °C and 200 rpm for 30 min, then filter-sterilized through a 0.22 μm microporous membrane. Briefly, 0.2 g of fresh mycelia was mixed with 10 mL of fresh sterile enzyme solution and digested at 30 °C with gentle shaking at 100 rpm for 2 h. The digested mixture was filtered through a six-layer sterile filter cloth, and the filtrate was centrifuged at 4000 rpm and 4 °C for 30 min to collect protoplasts. The sediments were fully washed with osmotic stabilizer to remove residual enzyme solution, re-centrifuged under the same conditions, and finally resuspended in STC buffer. The protoplast yield was quantified microscopically using a hemocytometer.

#### 2.3.2. Osmotic Stabilizers

Mycelia cultured for 72 h were harvested to evaluate the effects of different osmotic stabilizers on protoplast production. The enzymatic solutions were prepared separately with 0.7 M NaCl, 1.2 M KCl, or 1.2 M MgSO_4_ as osmotic stabilizers. Enzymatic digestion and protoplast collection were performed as described in Section 2.3.1. Subsequently, the protoplast yields for each treatment were counted using a hemocytometer under a light microscope.

#### 2.3.3. Enzyme Composition

Freshly harvested mycelia were thoroughly washed with an osmotic stabilizer and then treated with various enzyme preparations. To obtain high-yield and high-quality protoplasts, various single-enzyme and dual-enzyme combinations with different concentrations, including 20 mg/mL driselase (E1), 20 mg/mL lysing enzyme (E2), 20 mg/mL driselase + 20 mg/mL lysing enzyme (E3), 20 mg/mL driselase + 10 mg/mL lysing enzyme (E4), and 30 mg/mL driselase + 10 mg/mL lysing enzyme (E5). Cell wall digestion and protoplast collection were performed as described in Section 2.3.1. The final protoplast yield of each group was determined using a hemocytometer.

#### 2.3.4. Digestion Time and Shaking Speed

Enzymatic digestion was performed at different incubation durations (2 h, 3 h, and 5 h) and shaking speeds (100 rpm, 150 rpm, and 200 rpm) to improve protoplast yield further. Protoplast isolation and statistical analysis were conducted according to the protocol in Section 2.3.1, and protoplast yields for all treatments were observed and counted under a light microscope.

### 2.4. Screening of Protoplast Regeneration Medium

Different regeneration media supplemented with various osmotic stabilizers were screened to efficiently promote cell regeneration and normal colony growth of *protoplasts from I. robusta* and *I. vredehoekensi*. PDA and TB3 agar media were used as basal substrates, and five osmotic stabilizers (NaCl, KCl, sucrose, mannitol, and sorbitol) were separately added to the two basal media to prepare regeneration plates. Freshly isolated protoplasts harvested from prior isolation procedures were collected and adjusted to a concentration of 2 × 10^5^ CFU/mL using STC Buffer. After thorough mixing, 30 μL of protoplast suspension was evenly spread onto each regeneration medium plate, then incubated at 20 °C for 14 days. The colony number on each plate was counted, and the protoplast regeneration rate was calculated to assess regeneration efficiency across different regeneration media.

The formula for calculating the protoplast regeneration rate is as follows:

R = (C_1_ − C_2_)/C_3_ × 100

where R represents the protoplast regeneration rate (%), C_1_ represents the number of colonies formed on the regeneration medium, C_2_ represents the number of colonies formed on PDA medium (control), and C_3_ represents the total number of protoplasts.

### 2.5. PEG-Mediated Protoplast Transformation

Ten micrograms of plasmid DNA carrying the hygromycin B resistance gene were fully mixed with 200 μL of protoplast suspension at a concentration of 2 × 10^7^ CFU/mL, and the mixture was incubated on ice for 30 min. Subsequently, 1 mL of PTC Buffer was slowly added, followed by incubation on ice for another 30 min. Subsequently, the resulting mixture was transferred into 5 mL of TB3 liquid medium and cultured overnight at 20 °C with shaking at 100 rpm to facilitate protoplast recovery and initial cell wall regeneration. After incubation, the mixture was centrifuged at 4000 rpm for 10 min; part of the supernatant was removed, leaving approximately 1 mL of residual liquid to resuspend the precipitate fully. The resuspended suspension was blended with 20 mL of TB3 regeneration medium (containing 10 μg/mL hygromycin B) and poured into sterile Petri dishes to prepare plates. Plates were then cultured at 20 °C until visible colonies appeared. Single colonies were selected and sub-cultured on PDA medium (containing 25 μg/mL hygromycin B). For genetic stability verification, the candidate transformants were continuously sub-cultured for six consecutive generations. Strains with stable growth and normal morphological characteristics were considered positive transformants with stable GFP expression. An orthogonal experiment was designed with two key variables: PEG4000 concentration and STC Buffer type to optimize transformation efficiency further. Among them, three PEG4000 concentration gradients were set at 20%, 40%, and 60%, and three STC Buffer types were evaluated, including mannitol-based, sucrose-based, and sorbitol-based formulations.

### 2.6. Fluorescence Verification of Transformants

To verify successful plasmid transformation and heterologous expression, five randomly selected sub-cultured transformants were detected for *GFP* signals using a Zeiss inverted fluorescence microscope, with the wild-type strain as the blank control. Fresh mycelia were picked, placed on glass slides with a drop of sterile distilled water, and then covered with coverslips to prepare temporary slides for microscopic observation. The excitation and emission wavelengths for *GFP* were set at 488 nm and 510 nm, respectively. Visualization of green fluorescence signals in fungal mycelia. The detection of distinct green fluorescence indicates successful transformation and stable expression of the exogenous plasmid in the fungal strains.

### 2.7. Molecular Identification of Transformants

Genomic DNA of the wild-type and transformant strains was extracted using CTAB [11]. The integration of the *GFP* gene into the fungal genome was verified by PCR amplification using the following primers: GFP-F (5′-GATTGGAATGCATGGAGGAGT-3′) and GFP-R (5′-GCTTCTCGTTGGGGTCTTTG-3′). The wild-type strain was used as the negative control. The PCR reaction system was as follows: 15 μL of 2× EasyTaq^®^ PCR SuperMix (+dye), 1 μL of primer GFP-F, 1 μL of primer GFP-R, 1 μL of DNA template, and ddH_2_O to a final volume of 30 μL. The PCR reaction conditions were as follows: pre-denaturation at 94 °C for 3 min; denaturation at 94 °C for 30 s; annealing at 56 °C for 30 s; extension at 72 °C for 45 s (30 cycles); and final extension at 72 °C for 7 min. Amplified PCR products were detected using 1% agarose gel electrophoresis.

### 2.8. Pathogenicity Verification of Transformants

To evaluate whether the genetic transformants altered fungal virulence, pathogenicity testing was performed on transformants with wild-type-like morphology and stable, strong GFP fluorescence. Conidia were collected from the selected transformants and wild-type strains, and the final concentration of the spore suspensions was adjusted to 1 × 10^7^ CFU/mL with sterile distilled water. Healthy two-year-old American ginseng roots were used as inoculation materials. The roots were surface-sterilized by immersion in 75% ethanol for 2 min and in a 1% sodium hypochlorite (NaClO) solution for 10 min, rinsed thoroughly with sterile water, and then placed in sterile Petri dishes lined with sterile filter paper. Sterile inoculation needles were used to create wounds approximately 2 mm deep on the root surface, and 20 μL of spore suspension was applied at each wound site. Each root had three inoculation points, with five biological replicates per fungal strain. All treated roots were cultured at 20 °C for 10 days, after which disease symptoms caused by transformants and wild-type strains were compared.

### 2.9. Statistical Analysis

All experiments were performed in triplicate. Data are presented as mean ± standard deviation (SD). Statistical analysis was performed using Microsoft Excel, GraphPad Prism 9, and SPSS software (SPSS Inc., Version 19, Chicago, IL, USA). Before analysis of variance (ANOVA), the Shapiro–Wilk test was used to verify the normal distribution of each treatment, and Levene’s test was applied to test the homogeneity of variance. Two-way ANOVA was used to test main effects and interaction for two-factor factorial experiments in this study; after a significant interaction was observed, simple-effect pairwise comparisons were conducted via Fisher’s LSD test within each strain. Duncan’s multiple range test was used for one-way ANOVA.

## 3. Results

### 3.1. Screening of Hygromycin B Resistance Concentration

**Figure 1:**
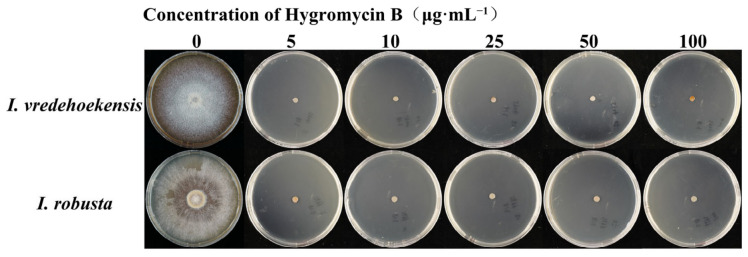
Susceptibility of *I. vredehoekensis* and *I. robusta* to hygromycin B (*n* = 3).

Hygromycin B is commonly used as a selectable marker in fungal genetic transformation methods. Determining the minimum inhibitory concentration (MIC) is a critical prerequisite for effectively distinguishing positive transformants from non-transformants, thereby eliminating interference from non-transformant contaminants and ensuring the accuracy of antibiotic screening results. Sensitivity assays with hygromycin B showed that *Ilyonectria* strains grew normally on PDA plates without hygromycin B supplementation. In contrast, the mycelial growth of both strains was completely suppressed when the hygromycin B concentration reached or exceeded 5 μg/mL (Figure 1). To further enhance the stringency of transformant screening and prevent false positives due to low antibiotic concentrations, 25 μg/mL hygromycin B was ultimately selected as the resistance screening concentration for transformants in subsequent genetic transformation experiments.

### 3.2. Optimization of Protoplast Preparation Conditions

#### 3.2.1. Effect of Mycelial Culture Time on Protoplast Preparation

**Figure 2:**
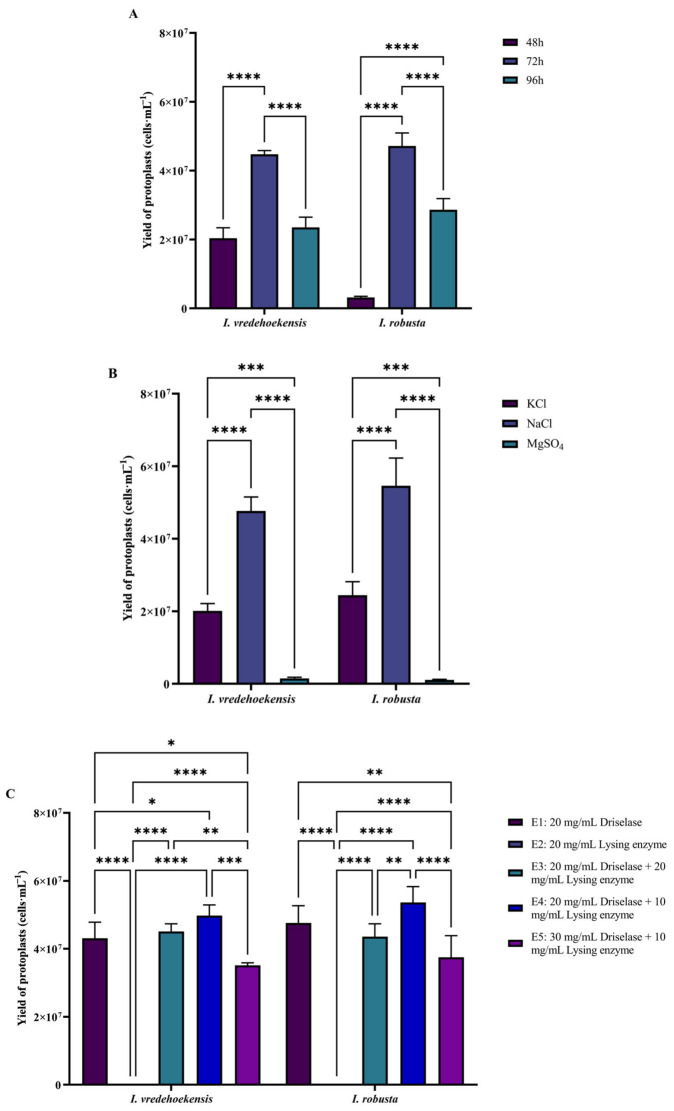

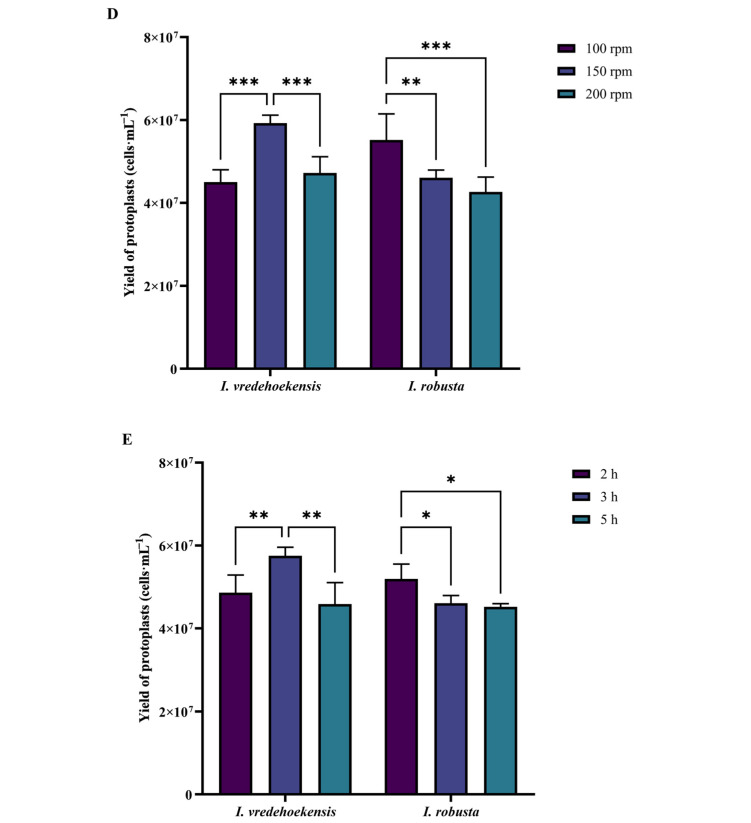
Protoplast preparation optimization in *I. vredehoekensis* and *I. robusta* (*n* = 3). Protoplast yields of *I. vredehoekensis* and *I. robusta* under different culture times (**A**), osmotic stabilizers (**B**), enzyme solutions (**C**), digestion agitation speeds (**D**), and digestion times (**E**). Data were shown as mean ± standard deviation (SD, *n* = 3), and error bars represented SD. Two-way ANOVA was used to assess the main effects of strain and treatment, as well as their interaction. The full ANOVA results are shown in Supplementary Tables S1–S10. The uncorrected Fisher’s LSD test was used for intra-strain multiple comparisons. Significance analysis between groups: * *p* < 0.05; ** *p* < 0.01; *** *p* < 0.001; **** *p* < 0.0001.

Mycelial biomass and physiological developmental stage directly affect the quality and yield of fungal protoplasts. Culture time assays showed that viable protoplasts could be successfully isolated from *I. vredehoekensis* and *I. robusta* at different culture times (48 h, 72 h, and 96 h), with a clear correlation between protoplast yield and culture time. Two-way ANOVA combined with Fisher’s LSD multiple comparison was performed for statistical analysis (Supplementary Tables S1 and S2). Before statistical analysis, the Shapiro–Wilk normality test indicated that all datasets were normally distributed (*p* > 0.05), meeting the prerequisite for two-way ANOVA. Protoplasts were obtained from mycelia cultured for 48 h in both fungi, but the yields differed significantly between species. The protoplast release from *I. vredehoekensis* reached 1.74 × 10^7^ CFU/mL, which was markedly higher than that from *I. robusta* (0.35 × 10^7^ CFU/mL). When the culture time was extended to 72 h, the protoplast yields of both strains peaked at 4.48 × 10^7^ and 4.72 × 10^7^ CFU/mL, respectively. Further prolongation of culture time led to a sharp reduction in protoplast production (Figure 2A).

#### 3.2.2. Effect of Osmotic Stabilizer Type on Protoplast Preparation

Osmotic stabilizers are mainly used to maintain the structural integrity of protoplasts. The three selected osmotic stabilizers (KCl, NaCl, and MgSO_4_) exerted significant effects on protoplast release. All experimental data conformed to the normal distribution, as verified by the Shapiro–Wilk normality test (*p* > 0.05, Supplementary Tables S3 and S4). When 0.7 M NaCl was used as the osmotic stabilizer, the protoplast yields of two fungi reached 4.77 × 10^7^ and 5.46 × 10^7^ CFU/mL, respectively, which were significantly higher than those obtained with KCl and MgSO_4_ (Figure 2B), indicating that 0.7 M NaCl was the optimal osmotic stabilizer for protoplast preparation of *I. vredehoekensis* and *I. robusta*.

#### 3.2.3. Effect of Enzyme Composition on Protoplast Preparation

A suitable cell wall-degrading enzymatic system is indispensable for efficient mycelial digestion of fungal cell walls and the acquisition of sufficient protoplasts. Driselase and lysing enzyme are complex enzyme mixtures containing cellulase, pectinase, protease, nuclease, and other enzymes that can effectively hydrolyze the major polysaccharide components of fungal cell walls, including β-1,3-glucan, cellulose, and xylan. The results revealed that the effects of single and combined application of the two enzymes differed significantly. Driselase single treatment (E1) effectively degraded the mycelia of both species, generating 4.31 × 10^7^ CFU/mL protoplasts for *I. vredehoekensis* and 4.76 × 10^7^ CFU/mL for *I. robusta*. In contrast, a single treatment with lysing enzyme (E2) failed to release protoplasts in either strain. Among the dual-enzyme combinations, the mixture of 20 mg/mL driselase + 10 mg/mL lysing enzyme (E4) showed the highest hydrolytic efficiency for both strains, and its protoplast yield was significantly higher than that of single-enzyme treatments (E1, E2) (*p* < 0.05). Under E4 treatment, the protoplast yields were 5.36 × 10^7^ CFU/mL for *I. vredehoekensis* and 4.97 × 10^7^ CFU/mL for *I. robusta*. Excessively elevated enzyme concentrations significantly reduced digestion efficiency (*p* < 0.05). For *I. vredehoekensis*, the E3 combination also produced a considerable protoplast yield, with no statistically significant difference compared with E4. For *I. robusta*, single Driselase treatment (E1) yielded more protoplasts (4.76 × 10^7^ CFU/mL) than compound enzyme groups E3 and E5 (Figure 2C). In summary, the combination of 20 mg/mL driselase and 10 mg/mL lysing enzyme (E4) exhibited optimal performance in both fungal strains.

#### 3.2.4. Effects of Digestion Speed and Duration on Protoplast Preparation

Moderate shaking speed during enzymatic digestion promotes adequate cell wall degradation while protecting the structural integrity of the protoplast. The speed of enzymatic digestion had distinct effects on protoplast yield in the two fungal species. For *I. robusta*, the maximum protoplast yield was obtained at 100 rpm, reaching 5.52 × 10^7^ CFU/mL, which was 1.20-fold and 1.30-fold higher than those at 150 rpm and 200 rpm, respectively. For *I. vredehoekensis*, the optimal shaking speed was 150 rpm, yielding 5.93 × 10^7^ CFU/mL, which significantly exceeded the values at 100 rpm and 200 rpm (Figure 2D).

In addition, the duration of digestion directly affects mycelial digestion and protoplast release efficiency. Within the three tested digestion durations (2, 3, and 5 h), the protoplast yield of *I. vredehoekensis* first increased and then decreased with the extension of incubation time, reaching a peak value of 5.75 × 10^7^ CFU/mL at 3 h and declining to 4.59 × 10^7^ CFU/mL after 5 h of digestion. For *I. robusta*, the highest yield of 5.19 × 10^7^ CFU/mL was achieved after 2 h of digestion, which was significantly higher than those at 3 h and 5 h (Figure 2E). Accordingly, the optimal digestion parameters were determined as 100 rpm and 2 h for *I. robusta*, and 150 rpm and 3 h for *I. vredehoekensis*.

**Figure 3:**
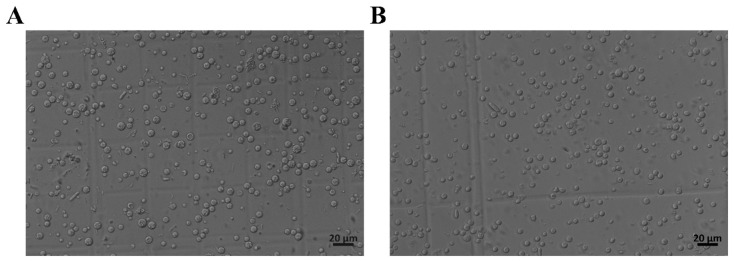
Morphology of protoplasts of *I. vredehoekensis* and *I. robusta*. (**A**) *I. vredehoekensis*; (**B**) *I. robusta*. Scale: 20 μm.

Taken together, based on the above experimental results, the optimal conditions for protoplast preparation of *I. vredehoekensis* and *I. robusta* were established. Briefly, the mycelia of the two fungal strains were cultured in YEPD liquid medium for 3 days. A mixed enzyme solution containing 20 mg/mL driselase and 10 mg/mL lysing enzyme (E4), dissolved in 0.7 M NaCl, was used as the enzymatic digestion system. Specifically, *I. robusta* was digested at 100 rpm for 3 h. *I vredehoekensis* was digested at 150 rpm for 2 h. Under these optimized conditions, the protoplast yields of *I. vredehoekensis* and *I. robusta* reached 5.93 × 10^7^ and 5.52 × 10^7^ CFU/mL, respectively (Figure 3).

### 3.3. Effect of Regeneration Media on Protoplast Regeneration

**Figure 4:**
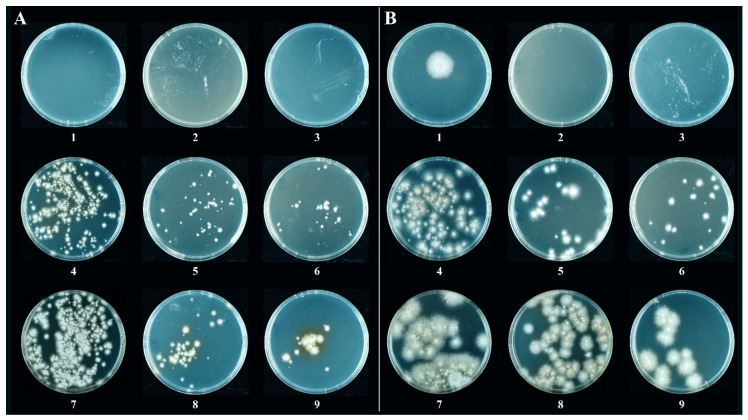
Effects of regeneration media on protoplast regeneration. (**A**) *I. vredehoekensis*; (**B**) *I. robusta*. (1) PDA, (2) PDA + NaCl, (3) PDA + KCl, (4) PDA + sucrose, (5) PDA + mannitol, (6) PDA + sorbitol, (7) TB3 + sucrose, (8) TB3 + mannitol, (9) TB3 + sorbitol.

The synergistic effects of osmotic stabilizers and nutrient substrates determine the efficiency of protoplast regeneration. A suitable regeneration medium can markedly promote cell wall regeneration efficiency and improve the survival rate of fungal protoplast colonies. The Shapiro–Wilk normality test and median-based Levene’s test of homogeneity of variance confirmed the feasibility of subsequent statistical analysis, and one-way ANOVA revealed highly significant differences in regeneration efficiency among treatments for both strains (*p* < 0.001; Supplementary Tables S11–S13). In this study, two basic media (PDA and TB3) were combined with five common osmotic stabilizers (NaCl, KCl, sucrose, mannitol, and sorbitol) to screen for optimal regeneration conditions. The results showed that different media combinations significantly affected protoplast regeneration efficiency in the two *Ilyonectria* strains. Overall, PDA supplemented with sucrose (PDA + sucrose) and TB3 supplemented with sucrose (TB3 + sucrose) were the most suitable regeneration media for protoplast regeneration in both fungal species. The regeneration efficiency of *I. vredehoekensis* was significantly higher than that of *I. robusta* under identical culture conditions (Table 1 and Figure 4). Among the five osmotic stabilizers, sucrose exhibited the strongest promoting effect on protoplast regeneration, which was significantly superior to mannitol, sorbitol, and inorganic salt stabilizers (NaCl and KCl). For *I. vredehoekensis*, TB3 medium supplemented with sucrose yielded the highest regeneration rate of 11.04%, significantly higher than those of all other treatment groups (*p* < 0.05). For *I. robusta*, both PDA + sucrose and TB3 + sucrose combinations exhibited better regeneration performance, with no significant difference between the two media. In contrast, inorganic osmotic stabilizers (NaCl and KCl) were unsuitable for protoplast regeneration in both fungi.

### 3.4. Optimization of PEG-Mediated Protoplast Transformation Conditions

Based on the previously optimized protoplast preparation and regeneration conditions of *I. vredehoekensis* and *I. robusta*, a L_9_(3^2^) orthogonal design was applied to optimize the PEG-CaCl_2_-mediated transformation system further. The results revealed that PEG4000 concentration and STC buffer type significantly affected transformation efficiency, and the two fungal species exhibited species-specific responses to these factors. As listed in Table 2, the transformation efficiency of *I. vredehoekensis* was more strongly influenced by the PEG4000 concentration than by the STC buffer type. The highest number of transformants was obtained under 40% PEG4000, which was remarkably higher than those obtained with 20% and 60% PEG4000. Meanwhile, the mannitol-formulated STC buffer (STC Buffer 1) provided the most favorable transformation condition. The optimal combination A_2_B_1_ (40% PEG4000 + STC Buffer 1) produced 38.67 ± 1.53 transformants, which significantly exceeded those of the other treatment groups. In comparison, *I. robusta* required more stringent conditions (Table 3). The PEG4000 concentration and STC buffer type exerted comparable effects on its transformation performance. Stable transformants were obtained only with the A_2_B_1_ combination (40% PEG4000 and STC Buffer 1), yielding an average of 12.70 ± 1.53 transformants, whereas no positive transformants were recovered from any other treatments. In summary, the combination of 40% PEG4000 and mannitol-based STC buffer (STC Buffer 1) achieved the best transformation performance for both fungi. An appropriate PEG concentration facilitates the efficient formation of exogenous DNA-protoplast complexes, while mannitol effectively maintains protoplast structural integrity during transformation. The synergistic effects of these two factors greatly facilitate successful genetic transformation, indicating that this optimized system has broad cross-species applicability within the genus *Ilyonectria*. However, the transformation efficiency of *I. vredehoekensis* was significantly higher than that of *I. robusta*, which may be attributed to inherent interspecific differences in the physiological sensitivity of fungal cells to the PEG-CaCl_2_-mediated transformation mechanism.

### 3.5. Fluorescence Evaluation of Transformants

**Figure 5:**
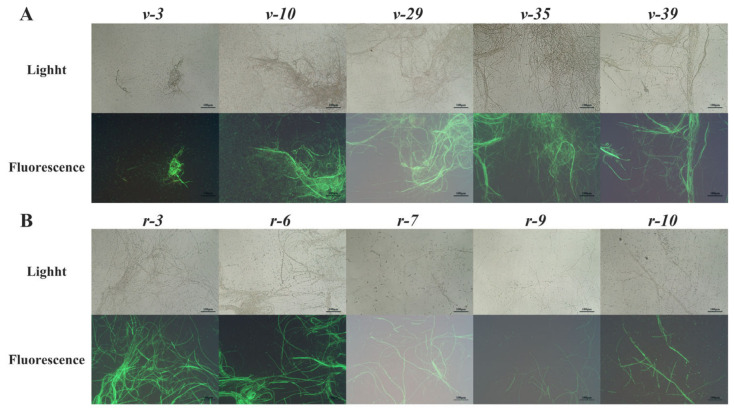
Fluorescence validation of transformants in two *Ilyonectria* species. (**A**) *I. vredehoekensis*; (**B**) *I. robusta*. v-3, v-10, v-29, v-35, and v-39 are GFP-tagged transformants of *I. vredehoekensis*; r-3, r-6, r-7, r-9, and r-10 are GFP-tagged transformants of *I. robusta*, respectively. The bars = 100 µm.

Candidate transformants with stable retained hygromycin resistance after six consecutive subcultures were selected for subsequent phenotypic and fluorescence identification. No significant differences in colonial morphology or growth rate were observed between the transformants and the corresponding wild-type strains, suggesting that the random insertion of exogenous genes did not adversely affect fungal growth phenotypes. Five transformants of *I. vredehoekensis* (v-3, v-10, v-29, v-35, and v-39) and five transformants of *I. robusta* (r-3, r-6, r-7, r-9, and r-10) were randomly chosen to examine GFP expression using a fluorescence microscope. Microscopic detection revealed that no green fluorescence signals were detected in the hyphae of wild-type strains. In contrast, strong, uniformly distributed green fluorescence was clearly observed in the mycelia of all transformants. These results confirmed that the exogenous *GFP* gene was successfully integrated into the fungal genome and stably expressed in both transformants (Figure 5). Minor differences in fluorescence intensity were observed among transformants, suggesting that variable insertion sites or different exogenous gene copy numbers may lead to variable gene expression levels.

### 3.6. PCR Verification of Transformants

**Figure 6:**
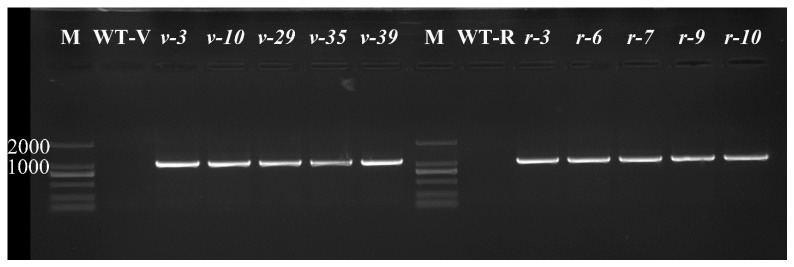
PCR identification of transformants in two *Ilyonectria* species. M: DNA marker; WT-V: wild-type strain of *I. vredehoekensis*; v-3, v-10, v-29, v-35, and v-39: transformants of *I. vredehoekensis*; WT-R: wild-type strain of *I. robusta*; r-3, r-6, r-7, r-9, and r-10: transformants of *I. robusta*.

PCR amplification was performed using *GFP*-specific primers to verify the genetic integration status of the fluorescence-positive transformants of *I. vredehoekensis* and *I. robusta*, with wild-type strains set as negative controls. Agarose gel electrophoresis results showed that all detected transformant strains amplified clear target bands of approximately 1000 bp. In contrast, no corresponding amplified fragments were detected in the two wild-type strains (Figure 6). The molecular detection results further confirmed that the exogenous *GFP* reporter gene was successfully and stably integrated into the genomes of the obtained *I. vredehoekensis* and *I. robusta* transformants, which was highly consistent with the fluorescence microscopy results. Collectively, this molecular evidence fully proved that the PEG-mediated genetic transformation system constructed in this study can efficiently realize stable genetic inheritance and continuous functional expression of exogenous genes in the two fungal strains.

### 3.7. Pathogenicity Evaluation of Positive Transformants

**Figure 7:**
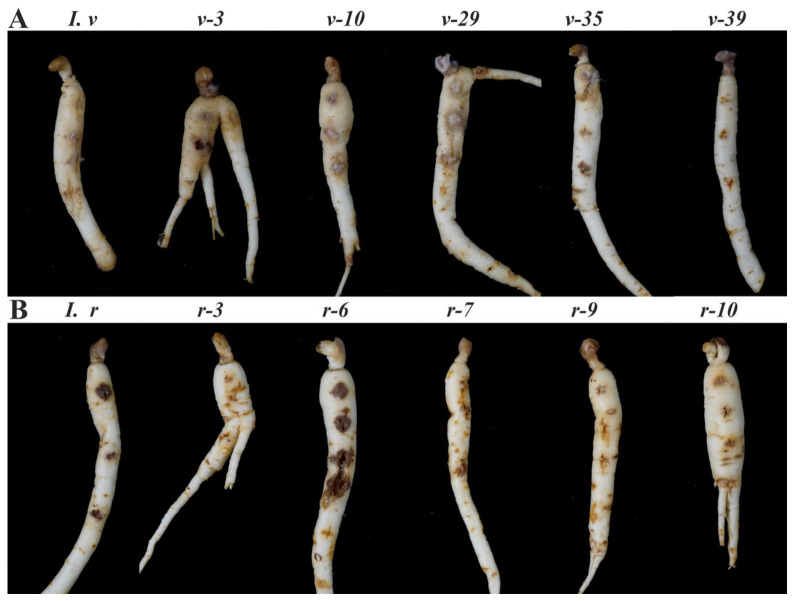
Pathogenicity of the transformants in two *Ilyonectria* species. (**A**) *I. vredehoekensis*; (**B**) *I. robusta. I.v* and *I.r* represent the wild-type strains of *I. vredehoekensis* and *I. robusta*, respectively; v-3, v-10, v-29, v-35, and v-39: transformants of *I. vredehoekensis*; r-3, r-6, r-7, r-9, and r-10: transformants of *I. robusta*. The figure shows the disease phenotypes observed on day 10 after inoculation.

Comparison of pathogenicity between wild-type strains and their corresponding transformants of *I. vredehoekensis* and *I. robusta* revealed evident interspecific divergence in virulence. Relative to *I. vredehoekensis*, *I. robusta* exhibited stronger pathogenicity and more rapid disease progression on American ginseng roots (Figure 7). At 4 days post-inoculation (dpi), typical brown spots appeared at the inoculation sites of the wild-type *I. robusta* strain and its transformants (r-6, r-7). The lesion area gradually expanded by 10 dpi, and root rot symptoms became progressively aggravated. In contrast, the wild-type *I. vredehoekensis* strain and its transformants (v-3, v-10, v-29, and v-35) did not develop obvious rot symptoms until 5 dpi, and visible white hyphae were observed surrounding the inoculation wounds at 10 dpi. The overall results of pathogenic phenotypes were highly consistent between transformants and their corresponding wild-type strains, indicating that exogenous *GFP* gene integration did not significantly alter fungal virulence. These results further confirmed that the established genetic transformation system can effectively maintain the original pathogenic characteristics of both strains, thereby providing a solid technical basis for subsequent studies of fluorescence labeling and pathogen-host interactions.

## 4. Discussion

Genetic transformation constitutes a fundamental approach for investigating gene function in fungi. In this study, we successfully established an efficient and stable genetic transformation method for two soil-borne pathogenic fungi, *I. vredehoekensis* and *I. robusta*, using the PEG-mediated protoplast transformation method (PMT). This work successfully addresses the existing lacunae in the technical literature concerning the genetic manipulation of these fungal species. As such, it provides a solid foundation for further investigations into their pathogenic mechanisms and disease control strategies. We also attempted *Agrobacterium tumefaciens*-mediated transformation (ATMT) in preliminary trials; however, no positive transformants were obtained. It was hypothesized that the observed failure could be attributed to intrinsic strain characteristics, structural features of the cell wall, or poor compatibility of the ATMT signaling pathway between these two fungi. Protoplast preparation and regeneration are essential prerequisites for fungal genetic transformation using the PMT method. In addition, optimizing PEG/CaCl_2_-mediated transformation conditions directly influences transformation efficiency and genetic stability.

In this study, we have established reliable protocols for protoplast preparation and regeneration of *I. vredehoekensis* and *I. robusta*. The selection of mycelial culture time is of paramount importance for obtaining the highest-quality mycelial material. Both *I. vredehoekensis* and *I. robusta* are slow-growing ascomycetes [24], and the optimal culture time for both strains was determined to be 72 h. This culture time was markedly longer than that of fast-growing *Fusarium oxysporum* (12 ~ 48 h) [32,35], but similar to that of the relatively slow-growing *Colletotrichum sublineola* (60 h) [34] and *Sphaerulina musiva* (72 h) [36]. The results indicate that the optimal mycelial culture time depends on the inherent growth rate of the fungal species. Slow-growing fungi require a longer culture period to reach a state conducive to enzymatic hydrolysis.

Fungal cell walls are structurally complex and predominantly composed of β-glucan, mannan oligosaccharides, and chitin, and the proportional composition of these components directly determines the efficiency of enzymatic digestion. The combined enzyme system of driselase and lysing enzymes contains multiple active components, including cellulase, pectinase, protease, nuclease, and various other hydrolases, which can synergistically degrade diverse fungal cell wall constituents. This dual enzyme mixture has been widely used for fungal cell wall digestion and is a common strategy to improve protoplast yield. In the present study, the optimal enzymatic hydrolysis conditions for the two *Ilyonectria* fungi were determined to be a combined treatment with 20 mg/mL driselase and 10 mg/mL lysing enzyme (E4). Notably, this optimal enzyme combination was consistent with that reported for *Verticillium dahliae* [37], suggesting that fungi share similar requirements for cell wall-degrading enzymes. However, obvious interspecific differences exist among fungi. Thus, it is speculated that these fungi possess similar proportional compositions of cell wall polysaccharides. Further results showed that a single treatment with 20 mg/mL driselase (E1) effectively degraded the mycelia of both *Ilyonectria* species, and the resulting protoplast yield was lower than that obtained with the E4 mixture, indicating that driselase plays a core role in the cell wall degradation of the two tested fungi. Driselase alone has also been proven effective for protoplast preparation of *F. oxysporum* [32] and *C. lindemuthianum* [38], further verifying its universal applicability in fungal cell wall digestion. In contrast, a single application of lysing enzyme (E2) failed to efficiently degrade the mycelial cell walls of the two *Ilyonectria* fungi. In contrast, *Aspergillus* strains exhibited pronounced cell wall-lysing effects [25]. Moreover, an excessive increase in driselase concentration within the compound enzyme system significantly reduced the enzymatic hydrolysis efficiency of both fungi. These divergent phenomena can be reasonably explained by structural differences in the outer-layer components of fungal cell walls, which govern the substrate specificity of digestive enzymes. Specifically, the outer cell walls of *F. oxysporum*, *V. dahlia,* and *C. lindemuthianum* are mainly composed of glycoproteins, while the inner rigid skeleton is composed of chitin and β-1,3-glucan [39,40,41]. Accordingly, cell wall disruption primarily relies on driselase, which is rich in proteases and chitinases. In contrast, *Aspergillus* fungi contain extremely low levels of cell wall glycoproteins, and their outer wall skeletons are mainly composed of specific glucans and galactomannans [42], enabling efficient cell wall degradation by a lysing enzyme rich in broad-spectrum glucanase. Based on the above results, we speculate that the outer cell wall of *Ilyonectria* fungi is mainly composed of glycoproteins, while the inner layer is structurally framed by chitin and β-1,3-glucan.

Enzymatic shaking speed and digestion duration mainly determine the contact efficiency between digestive enzymes and mycelial cell walls, thereby directly affecting protoplast isolation efficiency. In this study, the optimal shaking speed for *I. robusta* was 100 rpm, whereas *I. vredehoekensis* achieved the highest protoplast yield at 150 rpm. Compared with the previously reported rotation speed range of 60–140 rpm for most fungal enzymatic hydrolysis methods [43,44,45], which was presumed to be associated with the distinct degree of cell walls between the two species.

Marked variations in the optimal enzymatic digestion time have been reported among different fungi, ranging from 0.5 h to 12 h. Such divergence is closely linked to species-specific differences in cell wall composition, structural compactness, and the physiological status of mycelia. For instance, complete cell wall digestion can be achieved within 0.5–1 h in *Hirsutella sinensis* [46] and *Thecaphora thlaspeos* [47], implying relatively thin, loose cell wall structures that allow rapid infiltration and degradation by enzyme solutions. In contrast, sufficient enzymatic hydrolysis requires 4–12 h for *Hortaea wernecki* [48] and *Penicillium sclerotiorum* [49], likely due to their thickened, densely packed cell walls, which significantly hinder enzymatic reactions. Notably, most fungi possess intermediate cell wall structures between the two types above, with their optimal digestion time generally falling within 2–3 h. Consistently, the two *Ilyonectria* strains investigated in this study also conformed to this general rule.

Regeneration of protoplast cell walls is a key step in determining the overall success rate of fungal genetic transformation. Sugar alcohols are carbohydrates that are widely supplemented in regeneration media to maintain osmotic stability and facilitate cell wall reconstruction. In the present study, the sucrose-supplemented medium exhibited the best performance for protoplast regeneration in *I. vredehoekensis* and *I. robusta*, with regeneration efficiency significantly superior to that of the mannitol/sorbitol-supplemented media. Under these sucrose optimal conditions, the regeneration rate of *I. vredehoekensis* reached 11.04%, which was markedly higher than that of *I. robusta* (3.14%). We speculate that interspecific discrepancies are attributable to distinct transcriptional levels and catalytic efficiencies of cell wall biosynthesis-related enzymes, thereby enabling *I. vredehoekensis* to possess a stronger intrinsic capacity for cell wall recovery. Although viable regenerated colonies were obtained for both fungi, their regeneration rates were considerably lower than those reported for other fungi, such as *Eutypella* sp. (36%) [46] and *Sclerotiophoma versabilis* (90%) [47]. The results revealed that the protoplasts of the two tested fungi exhibited inherent difficulties in cell wall regeneration and low reconstruction efficiency, which may be attributed to their intrinsic physiological characteristics, complex cell wall architecture, and insufficient adaptation to current regeneration conditions. Further comparative analysis showed that *I. vredehoekensis* achieved a significantly higher regeneration rate in the TB3 + sucrose medium than in the PDA + sucrose medium. In contrast, no obvious difference was detected for *I. robusta* between the two media. This phenomenon reflects the intrinsic divergence in nutrient-use preferences, endogenous nutrient storage, and anabolic cell-wall metabolism among closely related fungal species.

The transformation efficiency of fungi in PEG/CaCl_2_-mediated fluorescent protein transformation is affected by PEG concentration. Our results revealed that *I. vredehoekensis* and *I. robusta*, two congeneric species, shared similar optimal transformation conditions with relatively minor specific differences, and that 40% PEG4000 was applied to both species. This result suggests that 40% PEG4000 can serve as a preferred reference concentration for establishing the protoplast transformation method for other *Ilyonectria* species. This consistency of transformation conditions among congeneric species is also found in *Fusarium*, a phylogenetically close genus to *Ilyonectria*. While a few species require higher PEG concentrations [32,48], 30–50% PEG4000 is the commonly used concentration for transforming most members of this genus, including *F. nematophilum*, *F. virguliforme*, and *F. graminearum* [49,50,51].

In addition, the type of sugar supplemented in the STC buffer also exerted a pronounced effect on protoplast survival and subsequent transformation efficiency. In this study, *I. vredehoekensis* exhibited broad adaptability to multiple sugar alcohol-based STC buffers. They can be successfully transformed under different osmotic conditions. In contrast, *I. robusta* exhibited strict specificity and yielded positive transformants only in mannitol-formulated STC buffer. Similar differential preferences for osmotic stabilizers have been widely documented across various fungal genera. For instance, multiple *Fusarium* and *Alternaria* species display distinct preferences for sugar alcohols during transformation. *F. virguliforme*, *F. oxysporum*, and *F. graminearum* can achieve high transformation efficiency in STC Buffer supplemented with sorbitol [32,43,51,52], whereas *F. rosicola* and *F. nematophilum* prefer sucrose as the osmotic stabilizer [49,53]. Additionally, *A. alternata* adapts well to both sucrose and sorbitol environments [54], whereas *A. solani* depends on mannitol for successful transformation [55]. Such interspecific divergence is likely attributable to inherent differences in plasma membrane permeability, regulation of intracellular osmotic homeostasis, and cell wall reconstruction metabolic pathways among fungal species. Based on the above findings, mannitol-based STC buffer is prioritized for protoplast transformation of other *Ilyonectria* strains. If the initial transformation outcome is unsatisfactory, supplementary optimization with sucrose or sorbitol can be performed to improve the experimental performance.

## 5. Conclusions

In this study, efficient protoplast preparation and stable PEG-mediated genetic transformation methods were successfully established for *I. vredehoekensis* and *I. robusta*, providing a solid technical basis for future functional gene research in *Ilyonectria* fungi. The overall optimal parameters were summarized as follows: a composite enzyme system consisting of 20 mg/mL driselase and 10 mg/mL lysing enzyme, together with 0.7 M NaCl as the optimal osmotic stabilizer. Under these conditions, highly active and high-quality protoplasts were obtained, with yields reaching 5.52 × 10^7^ CFU/mL for *I. vredehoekensis* (digestion at 150 rpm for 3 h) and 5.75 × 10^7^ CFU/mL for *I. robusta* (digestion at 100 rpm for 2 h). In the genetic transformation step, 40% PEG4000 combined with mannitol-formulated STC Buffer achieved the highest transformation efficiency. The protocol not only enables reliable investigations into functional genes, pathogenic mechanisms, and host-fungus interactions in *I. vredehoekensis* and *I. robusta*, but also holds considerable theoretical and practical implications for improving molecular genetic approaches across the genus *Ilyonectria*.

## Figures and Tables

**Table 1:** Effects of regeneration media on protoplast regeneration in two *Ilyonectria* fungi (*n* = 3).

Regeneration Media	Protoplast Regeneration Rate (%)	
	*I. vredehoekensis*	*I. robusta*
PDA	control	control
PDA + NaCl	0 ± 0.02 d	0 ± 0 c
PDA + KCl	0 ± 0 d	0 ± 0.06 c
PDA + sucrose	7.97 ± 1.42 b	3.14 ± 0.53 a
PDA + mannitol	1.99 ± 0.49 c	1.33 ± 0.70 b
PDA + sorbitol	2.17 ± 0.30 c	1.44 ± 0.92 b
TB3 + sucrose	11.04 ± 1.00 a	2.90 ± 0.19 a
TB3 + mannitol	1.73 ± 0.90 c	2.60 ± 0.23 a
TB3 + sorbitol	0.61 ± 0.02 d	0.64 ± 0.23 bc

Data are presented as mean values ± standard deviation (*n* = 3). Normality was assessed using the Shapiro–Wilk test, and the homogeneity of variance was verified using the media-based Levene’s test. A few of the treatments exhibited mild deviation from the normal distribution. Given that one-way ANOVA is robust to slight non-normality with small sample sizes, we adopted parametric statistical analysis. A one-way ANOVA followed by Duncan’s multiple range test was performed. Different letters indicate a significant difference among treatments at *p* < 0.05. Supplementary Tables S11–S13 provide detailed outputs of the normality test, homogeneity test, and ANOVA analysis.

**Table 2:** Optimization of PEG-CaCl_2_ mediated conditions for *I. vredehoekensis* (*n* = 3).

	A: PEG Concentration (%)	B: STC Buffer	Number of Transformants (Mean ± SD)
1	20	1	0 ± 0
2	20	2	0 ± 0
3	20	3	0 ± 0
4	40	1	38.67 ± 1.53
5	40	2	1.00 ± 1.00
6	40	3	1.33 ± 0.58
7	60	1	0.67 ± 0.58
8	60	2	0 ± 0
9	60	3	0 ± 0

**Table 3:** Optimization of PEG-CaCl_2_ mediated conditions for *I. robusta* (*n* = 3).

	A: PEG Concentration (%)	B: STC Buffer	Number of Transformants (Mean ± SD)
1	20	1	0 ± 0
2	20	2	0 ± 0
3	20	3	0 ± 0
4	40	1	12.70 ± 1.53
5	40	2	0 ± 0
6	40	3	0 ± 0
7	60	1	0 ± 0
8	60	2	0 ± 0
9	60	3	0 ± 0

## Data Availability

Data sharing is not applicable to this article.

## Supplementary Materials

The following supporting information can be downloaded at: https://www.mdpi.com/article/10.3390/jof12070488/s1. Table S1: Analysis of variance for protoplast yields of two *Ilyonectria* strains under different culture time treatments (*n* = 3); Table S2: Fisher’s LSD multiple comparisons of protoplast yields in two *Ilyonectria* strains under different culture time treatments (*n* = 3).; Table S3: Analysis of variance for protoplast yields of two *Ilyonectria* strains under different osmotic stabilizer treatments (*n* = 3); Table S4: Fisher’s LSD multiple comparisons of protoplast yields in two *Ilyonectria* strains under different osmotic stabilizer treatments (*n* = 3); Table S5: Analysis of variance for protoplast yields of two *Ilyonectria* strains under different enzyme combination treatments (*n* = 3); Table S6: Fisher’s LSD multiple comparisons of protoplast yields in two *Ilyonectria* strains under different enzyme combination treatments (*n* = 3); Table S7: Analysis of variance for protoplast yields of two *Ilyonectria* strains under different shaking speed treatments (*n* = 3); Table S8: Fisher’s LSD multiple comparisons of protoplast yields in two *Ilyonectria* strains under different shaking speed treatments (*n* = 3); Table S9: Analysis of variance for protoplast yields of two *Ilyonectria* strains under different digestion duration treatments (*n* = 3); Table S10: Fisher’s LSD multiple comparisons of protoplast yields in two *Ilyonectria* strains under different digestion duration treatments (*n* = 3); Table S11: Shapiro–Wilk normality test for protoplast regeneration rate in two *Ilyonectria* strains (*n* = 3); Table S12: Levene’s test for homogeneity of variance across different regeneration media in two *Ilyonectria* strains (*n* = 3); Table S13: The One-way ANOVA results of protoplast regeneration rate under different regeneration media in two *Ilyonectria* strains (*n* = 3).
