# Supplementary material for: Establishment of Protoplast Preparation and Genetic Transformation Methods in Two Ilyonectria Species
Source: J Fungi (Basel). 2026 Jul 2;12(7):488. doi: 10.3390/jof12070488 (PMC13413419; doi:10.3390/jof12070488)
Supplement: Supplementary file 1 [file jof-12-00488-s001.zip › jof-4379467-supplementary.pdf]

**Supplementary Table S1.** Analysis of variance for protoplast yields of two *Ilyonectria* strains under different culture time treatments ($n$ = 3).

| **Source of variation** | **Sum of squares (SS)** | **Degrees of freedom (DF)** | **Mean square (MS)** | **F value (DFn, DFd)** | ***P* value** | **Significance** |
|---|---|---|---|---|---|---|
| Strain (main effect) | $4.69 \times 10^{12}$ | 1 | $4.69 \times 10^{12}$ | F (1, 4) = 6.087 | 0.0692 | ns |
| Culture time (main effect of culture duration) | $3.54 \times 10^{15}$ | 2 | $1.77 \times 10^{15}$ | F (2, 8) = 245.8 | < 0.0001 | **** |
| Culture time × Strain (interaction) | $4.44 \times 10^{14}$ | 2 | $2.22 \times 10^{14}$ | F (2, 8) = 30.85 | 0.0002 | *** |
| Residual (error) | $5.75 \times 10^{12}$ | 8 | $7.20 \times 10^{11}$ | - | - | - |

The experiment included two *Ilyonectria* strains with three culture time treatments, and three independent biological replicates per group ($n$ = 3). Prior to two-way ANOVA, Shapiro-Wilk normality test was carried out on each group. All datasets satisfied the normal distribution assumption ($P > 0.05$). Additional skewness tests could not be performed due to the limited sample size. Two-way ANOVA was used to analyze the main effects of strain and culture time, as well as their interaction. Significance analysis between groups: ns, not significant; *, $P < 0.05$; **, $P < 0.01$; ***, $P < 0.001$; ****, $P < 0.0001$.

**Supplementary Table S2.** Fisher's LSD multiple comparisons of protoplast yields in two *Ilyonectria* strains under different culture time treatments ($n$ = 3).

| Strain | Culture time (h) | Mean yield ± SD (× $10^7$ cells · $mL^{-1}$) | Biological replicates (n) | Comparison | Mean difference | Fisher's LSD *P*-value | Significance |
|---|---|---|---|---|---|---|---|
| *I. vredehoekensis* | 48 | 2.04 ± 0.11 | 3 | 48 h vs 72 h | $-2.44 \times 10^7$ | < 0.0001 | **** |
| | 72 | 4.48 ± 0.11 | 3 | 48 h vs 96 h | $-3.17 \times 10^6$ | 0.1862 | ns |
| | 96 | 2.35 ± 0.30 | 3 | 72 h vs 96 h | $2.12 \times 10^7$ | < 0.0001 | **** |
| *I. robusta* | 48 | 0.32 ± 0.003 | 3 | 48 h vs 72 h | $-4.40 \times 10^7$ | < 0.0001 | **** |
| | 72 | 4.72 ± 0.38 | 3 | 48 h vs 96 h | $-2.55 \times 10^7$ | < 0.0001 | **** |
| | 96 | 2.87 ± 0.32 | 3 | 72 h vs 96 h | $1.85 \times 10^7$ | < 0.0001 | **** |

Pairwise comparisons were only conducted between different culture time treatments within the same strain, and cross-strain comparisons were not performed. Simple-effect analyses were implemented after a significant interaction was observed in the two-way ANOVA. Uncorrected Fisher's LSD test was used for intra-strain multiple comparisons. Significance analysis between groups: ns, not significant; *, $P < 0.05$; **, $P < 0.01$; ***, $P < 0.001$; ****, $P < 0.0001$.

**Supplementary Table S3.** Analysis of variance for protoplast yields of two *Ilyonectria* strains under different osmotic stabilizer treatments ($n$ = 3).

| Source of variation | Sum of squares (SS) | Degrees of freedom (DF) | Mean square (MS) | F value (DFn, DFd) | $P$ value | Significance |
|---|---|---|---|---|---|---|
| Strain (main effect) | $7.52 \times 10^{15}$ | 2 | $3.76 \times 10^{15}$ | F (2, 8) = 190.4 | < 0.0001 | **** |
| Osmotic stabilizer (main effect of culture duration) | $5.90 \times 10^{12}$ | 1 | $5.90 \times 10^{12}$ | F (1, 4) = 9.119 | 0.0392 | * |
| Osmotic stabilizer × Strain (interaction) | $4.21 \times 10^{12}$ | 2 | $2.10 \times 10^{12}$ | F (2, 8) = 1.065 | 0.389 | ns |
| Residual (error) | $1.58 \times 10^{14}$ | 8 | $1.98 \times 10^{13}$ | - | - | - |

The experiment included two *Ilyonectria* strains with three osmotic stabilizer treatments, and three independent biological replicates per group ($n$ = 3). Prior to two-way ANOVA, Shapiro-Wilk normality test was carried out on each group. All datasets satisfied the normal distribution assumption ($P > 0.05$). Additional skewness tests could not be performed due to the limited sample size. Two-way ANOVA was used to analyze the main effects of strain and osmotic stabilizer treatments, as well as their interaction. Significance analysis between groups: ns, not significant; *, $P < 0.05$; **, $P < 0.01$; ***, $P < 0.001$; ****, $P < 0.0001$.

**Supplementary Table S4.** Fisher's LSD multiple comparisons of protoplast yields in two *Ilyonectria* strains under different osmotic stabilizer treatments ($n$ = 3).

| Strain | Osmotic stabilizer | Mean yield ± SD (× $10^7$ cells · $mL^{-1}$) | Biological replicates (n) | Comparison | Mean difference | Fisher's LSD *P*-value | Significance |
|---|---|---|---|---|---|---|---|
| *I. vredehoekensis* | NaCl | 4.77 ± 0.39 | 3 | KCl vs $MgSO_4$ | -3.28 × $10^7$ | 0.0009 | *** |
| | KCl | 2.01 ± 0.20 | 3 | KCl vs NaCl | -2.76 × $10^7$ | < 0.0001 | **** |
| | $MgSO_4$ | 0.15 ± 0.06 | 3 | NaCl vs $MgSO_4$ | 5.27 × $10^6$ | < 0.0001 | **** |
| *I. robusta* | NaCl | 5.46 ± 0.77 | 3 | KCl vs $MgSO_4$ | -4.41 × $10^7$ | 0.0002 | *** |
| | KCl | 2.45 ± 0.37 | 3 | KCl vs NaCl | -3.02 × $10^7$ | < 0.0001 | **** |
| | $MgSO_4$ | 0.11 ± 0.02 | 3 | NaCl vs $MgSO_4$ | 1.37 × $10^7$ | < 0.0001 | **** |

Pairwise comparisons were only conducted between different osmotic stabilizer treatments within the same strain, and cross-strain comparisons were not performed. Simple-effect analyses were implemented after a significant interaction was observed in the two-way ANOVA. Uncorrected Fisher's LSD test was used for intra-strain multiple comparisons. Significance analysis between groups: ns, not significant; *, $P < 0.05$; **, $P < 0.01$; ***, $P < 0.001$; ****, $P < 0.0001$.

**Supplementary Table S5.** Analysis of variance for protoplast yields of two *Ilyonectria* strains under different enzyme combination treatments ($n$ = 3).

| Source of variation | Sum of squares (SS) | Degrees of freedom (DF) | Mean square (MS) | F value (DFn, DFd) | $P$ value | Significance |
|---|---|---|---|---|---|---|
| Strain (main effect) | $2.57 \times 10^{12}$ | 1 | $2.57 \times 10^{12}$ | F (1, 4) = 1.550 | 0.2811 | ns |
| Enzyme combination treatments (main effect of culture duration) | $1.02 \times 10^{16}$ | 4 | $2.54 \times 10^{15}$ | F (4, 16) = 188.9 | < 0.0001 | **** |
| Enzyme combination treatments × Strain (interaction) | $3.92 \times 10^{11}$ | 4 | $9.79 \times 10^{10}$ | F (4, 16) = 0.7275 | 0.5861 | ns |
| Residual (error) | $2.15 \times 10^{14}$ | 16 | $1.35 \times 10^{13}$ | - | - | - |

The experiment included two *Ilyonectria* strains with five enzyme combination treatments, and three independent biological replicates per group ($n$ = 3). Prior to two-way ANOVA, Shapiro-Wilk normality test was carried out on each group. All datasets satisfied the normal distribution assumption ($P > 0.05$). Additional skewness tests could not be performed due to the limited sample size. Two-way ANOVA was used to analyze the main effects of strain and enzyme combination treatments, as well as their interaction. Significance analysis between groups: ns, not significant; *, $P < 0.05$; **, $P < 0.01$; ***, $P < 0.001$; ****, $P < 0.0001$.

**Supplementary Table S6.** Fisher's LSD multiple comparisons of protoplast yields in two *Ilyonectria* strains under different enzyme combination treatments ($n$ = 3).

| Strain | Enzyme combination | Mean yield ± SD ($\times 10^7$ cells · $mL^{-1}$) | Biological replicates (n) | Comparison | Mean difference | Fisher's LSD *P*-value | Significance |
|---|---|---|---|---|---|---|---|
| *I. vredehoekensis* | E1 | 4.31 ± 0.47 | 3 | E1 vs E2 | $4.31 \times 10^7$ | < 0.0001 | **** |
| | | | | E1 vs E3 | $-2.00 \times 10^6$ | 0.5139 | ns |
| | E2 | 0.00 ± 0.003 | 3 | E1 vs E4 | $-6.67 \times 10^6$ | 0.0408 | * |
| | | | | E1 vs E5 | $7.95 \times 10^6$ | 0.0173 | * |
| | E3 | 4.51 ± 0.23 | 3 | E2 vs E3 | $-4.51 \times 10^7$ | < 0.0001 | **** |
| | | | | E2 vs E4 | $-4.98 \times 10^7$ | < 0.0001 | **** |
| | E4 | 4.98 ± 0.32 | 3 | E2 vs E5 | $-3.51 \times 10^7$ | < 0.0001 | **** |
| | | | | E3 vs E4 | $-4.67 \times 10^6$ | 0.1388 | ns |
| | E5 | 3.52 ± 0.07 | 3 | E3 vs E5 | $9.95 \times 10^6$ | 0.0043 | ** |
| | | | | E4 vs E5 | $1.46 \times 10^7$ | 0.0002 | *** |
| *I. robusta* | E1 | 4.76 ± 0.51 | 3 | E1 vs E2 | $4.76 \times 10^7$ | < 0.0001 | **** |
| | | | | E1 vs E3 | $4.05 \times 10^6$ | 0.1952 | ns |
| | E2 | 0.01 ± 0.003 | 3 | E1 vs E4 | $-6.02 \times 10^6$ | 0.0618 | ns |
| | | | | E1 vs E5 | $1.01 \times 10^7$ | 0.0038 | ** |
| | E3 | 4.36 ± 0.38 | 3 | E2 vs E3 | $-4.35 \times 10^7$ | < 0.0001 | **** |
| | | | | E2 vs E4 | $-5.36 \times 10^7$ | < 0.0001 | **** |
| | E4 | 5.36 ± 0.47 | 3 | E2 vs E5 | $-3.74 \times 10^7$ | < 0.0001 | **** |
| | | | | E3 vs E4 | $-1.01 \times 10^7$ | 0.004 | ** |
| | E5 | 3.75 ± 0.64 | 3 | E3 vs E5 | $6.07 \times 10^6$ | 0.0599 | ns |
| | | | | E4 vs E5 | $1.61 \times 10^7$ | < 0.0001 | **** |

Pairwise comparisons were only conducted between different enzyme combination treatments within the same strain, and cross-strain comparisons were not performed. Simple-effect analyses were implemented after a significant interaction was observed in the two-way ANOVA. Uncorrected Fisher's LSD test was used for intra-strain multiple comparisons. Significance analysis between groups: ns, not significant; *, $P < 0.05$; **, $P < 0.01$; ***, $P < 0.001$; ****, $P < 0.0001$.

**Supplementary Table S7.** Analysis of variance for protoplast yields of two *Ilyonectria* strains under different shaking speed treatments ($n$ = 3).

| **Source of variation** | **Sum of squares (SS)** | **Degrees of freedom (DF)** | **Mean square (MS)** | **F value (DFn, DFd)** | ***P* value** | **Significance** |
|---|---|---|---|---|---|---|
| Strain (main effect) | $1.19 \times 10^{14}$ | 4 | $2.97 \times 10^{13}$ | F (4, 8) = 4.735 | 0.0297 | * |
| Shaking speed (main effect of culture duration) | $1.86 \times 10^{14}$ | 2 | $9.30 \times 10^{13}$ | F (2, 8) = 14.82 | 0.002 | ** |
| Shaking speed × Strain (interaction) | $4.18 \times 10^{14}$ | 2 | $2.09 \times 10^{14}$ | F (2, 8) = 33.33 | 0.0001 | *** |
| Residual (error) | $5.02 \times 10^{13}$ | 8 | $6.27 \times 10^{12}$ | - | - | - |

The experiment included two *Ilyonectria* strains with three shaking speed treatments, and three independent biological replicates per group ($n$ = 3). Prior to two-way ANOVA, Shapiro-Wilk normality test was carried out on each group. All datasets satisfied the normal distribution assumption ($P > 0.05$). Additional skewness tests could not be performed due to the limited sample size. Two-way ANOVA was used to analyze the main effects of strain and shaking speed treatments, as well as their interaction. Significance analysis between groups: ns, not significant; *, $P < 0.05$; **, $P < 0.01$; ***, $P < 0.001$; ****, $P < 0.0001$.

**Supplementary Table S8.** Fisher's LSD multiple comparisons of protoplast yields in two *Ilyonectria* strains under different shaking speed treatments ($n$ = 3).

| Strain | Shaking speed (rpm) | Mean yield ± SD (× $10^7$ cells · $mL^{-1}$) | Biological replicates (n) | Comparison | Mean difference | Fisher's LSD *P*-value | Significance |
|---|---|---|---|---|---|---|---|
| *I. vredehoekensis* | 100 | 4.77 ± 0.39 | 3 | KCl vs $MgSO_4$ | -3.28 × $10^7$ | 0.0009 | *** |
| | 150 | 2.01 ± 0.20 | 3 | KCl vs NaCl | -2.76 × $10^7$ | < 0.0001 | **** |
| | 200 | 0.15 ± 0.06 | 3 | NaCl vs $MgSO_4$ | 5.27 × $10^6$ | < 0.0001 | **** |
| *I. robusta* | 100 | 5.46 ± 0.77 | 3 | KCl vs $MgSO_4$ | -4.41 × $10^7$ | 0.0002 | *** |
| | 150 | 2.45 ± 0.37 | 3 | KCl vs NaCl | -3.02 × $10^7$ | < 0.0001 | **** |
| | 200 | 0.11 ± 0.02 | 3 | NaCl vs $MgSO_4$ | 1.37 × $10^7$ | < 0.0001 | **** |

Pairwise comparisons were only conducted between different shaking speed treatments within the same strain, and cross-strain comparisons were not performed. Simple-effect analyses were implemented after a significant interaction was observed in the two-way ANOVA. Uncorrected Fisher's LSD test was used for intra-strain multiple comparisons. Significance analysis between groups: ns, not significant; *, $P < 0.05$; **, $P < 0.01$; ***, $P < 0.001$; ****, $P < 0.0001$.

**Supplementary Table S9.** Analysis of variance for protoplast yields of two *Ilyonectria* strains under different digestion duration treatments (*n* = 3).

| **Source of variation** | **Sum of squares (SS)** | **Degrees of freedom (DF)** | **Mean square (MS)** | **F value (DFn, DFd)** | ***P* value** | **Significance** |
| --- | --- | --- | --- | --- | --- | --- |
| Strain (main effect) | 3.92 ×1012 | 1 | 3.92 × 1012 | F (1, 4) = 2.518 | 0.1878 | ns |
| Digestion duration (main effect of culture duration) | 1.28 ×1014 | 2 | 6.40 ×1013 | F (2, 8) = 7.276 | 0.0158 | * |
| Digestion duration × Strain (interaction) | 1.75 × 1014 | 2 | 8.75 ×1013 | F (2, 8) = 9.961 | 0.0067 | ** |
| Residual (error) | 7.03 ×1013 | 8 | 8.79 ×1012 | - | - | - |

The experiment included two *Ilyonectria* strains with three digestion duration treatments, and three independent biological replicates per group ($n = 3$). Prior to two-way ANOVA, Shapiro-Wilk normality test was carried out on each group. All datasets satisfied the normal distribution assumption ($P > 0.05$). Additional skewness tests could not be performed due to the limited sample size. Two-way ANOVA was used to analyze the main effects of strain and digestion duration treatments, as well as their interaction. Significance analysis between groups: ns, not significant; *, $P < 0.05$; **, $P < 0.01$; ***, $P < 0.001$; ****, $P < 0.0001$.

**Supplementary Table S10.** Fisher's LSD multiple comparisons of protoplast yields in two *Ilyonectria* strains under different digestion duration treatments ($n$ = 3).

| Strain | Digestion duration (h) | Mean yield ± SD (× 10$^7$ cells · mL$^{-1}$) | Biological replicates (n) | Comparison | Mean difference | Fisher's LSD *P*-value | Significance |
|---|---|---|---|---|---|---|---|
| *I. vredehoekensis* | 2 | 4.86 ± 0.43 | 3 | 2 h vs 3 h | -8.90 × 10$^6$ | 0.0062 | ** |
| | 3 | 5.75 ± 0.21 | 3 | 2 h vs 5 h | 2.75 × 10$^6$ | 0.2888 | ns |
| | 5 | 4.59 ± 0.52 | 3 | 3 h vs 5 h | 1.17 × 10$^7$ | 0.0013 | ** |
| *I. robusta* | 2 | 5.19 ± 0.36 | 3 | 2 h vs 3 h | 5.87 × 10$^6$ | 0.0416 | * |
| | 3 | 4.61 ± 0.19 | 3 | 2 h vs 5 h | 6.73 × 10$^6$ | 0.0239 | * |
| | 5 | 4.52 ± 0.08 | 3 | 3 h vs 5 h | 8.67 × 10$^5$ | 0.7296 | ns |

Pairwise comparisons were only conducted between different digestion duration treatments within the same strain, and cross-strain comparisons were not performed. Simple-effect analyses were implemented after a significant interaction was observed in the two-way ANOVA. Uncorrected Fisher's LSD test was used for intra-strain multiple comparisons. Significance analysis between groups: ns, not significant; *, $P < 0.05$; **, $P < 0.01$; ***, $P < 0.001$; ****, $P < 0.0001$.

**Supplementary Table S11.** Shapiro-Wilk normality test for protoplast regeneration rate in two *Ilyonectria* strains (*n* = 3).

| Strain | Source of variation | W statistic | *P*-value | Normality |
|---|---|---|---|---|
| *I. vredehoekensis* | PDA + NaCl | - | - | Zero values, not tested |
| | PDA + KCl | - | - | Zero values, not tested |
| | PDA + Sucrose | 0.944 | 0.543 | Normal |
| | PDA + Mannitol | 0.832 | 0.194 | Normal |
| | PDA + Sorbitol | 0.982 | 0.744 | Normal |
| | TB3 + Sucrose | 0.999 | 0.956 | Normal |
| | TB3 + Mannitol | 0.889 | 0.352 | Normal |
| | TB3 + Sorbitol | 0.750 | < 0.05 | Non-normal |
| *I. robusta* | PDA + NaCl | - | | Zero values, not tested |
| | PDA + KCl | 0.750 | < 0.05 | Non-normal |
| | PDA + Sucrose | 0.959 | 0.609 | Normal |
| | PDA + Mannitol | 0.862 | 0.274 | Normal |
| | PDA + Sorbitol | 0.932 | 4.495 | Normal |
| | TB3 + Sucrose | 0.981 | 0.734 | Normal |
| | TB3 + Mannitol | 0.750 | < 0.05 | Non-normal |
| | TB3 + Sorbitol | 0.913 | 0.428 | Normal |

Shapiro-Wilk normality test was performed for each treatment group. Three groups with zero regeneration values were not subjected to normality analysis. A small number of groups showed mild non-normal distribution due to skewed replicate data.

**Supplementary Table S12.** Levene's test for homogeneity of variance across different regeneration media in two *Ilyonectria* strains ($n$ = 3).

| Strain | Levene's method | F value | $df_1$ | $df_2$ | $P$-value |
|---|---|---|---|---|---|
| *I. vredehoekensis* | Based on mean | 4.367 | 7 | 16 | 0.007 |
| | Based on median | 1.435 | 7 | 16 | 0.259 |
| *I. robusta* | Based on mean | 5.006 | 7 | 16 | 0.004 |
| | Based on median | 1.042 | 7 | 16 | 0.441 |

Levene's test was used to evaluate homogeneity of variance. The conventional mean-based Levene test indicated unequal variance, while the median-based Levene test, which is robust to skewed data, confirmed homogeneous variance across all treatment groups ($P > 0.05$). We adopted the result from the median-based Levene test for subsequent ANOVA analysis.

**Supplementary Table S13.** One-way ANOVA results of protoplast regeneration rate under different regeneration media in two *Ilyonectria* strains ($n$ = 3).

| Strain | Source of variation | Sum of squares (SS) | Degrees of freedom (DF) | Mean square (MS) | F value (DFn, DFd) | Significance |
|---|---|---|---|---|---|---|
| *I. vredehoekensis* | Between Groups | 348.527 | 7 | 49.79 | F (7, 16) = 95.274 | < 0.001 |
| | Within Groups | 8.361 | 16 | 0.523 | - | - |
| | Total | 356.888 | 23 | - | - | - |
| *I. robusta* | Between Groups | 33.414 | 7 | 4.773 | F (7, 16) = 21.828 | < 0.001 |
| | Within Groups | 3.499 | 16 | 0.219 | - | - |
| | Total | 36.913 | 23 | - | - | - |

One-way analysis of variance (ANOVA) was conducted separately for the two *Ilyonectria* strains. Significant differences were observed among different regeneration media for both strains ($P < 0.001$).
